# Effects of Long-Term Denosumab on Bone Histomorphometry and Mineralization in Women With Postmenopausal Osteoporosis

**DOI:** 10.1210/jc.2017-02669

**Published:** 2018-04-16

**Authors:** David W Dempster, Jacques P Brown, Astrid Fahrleitner-Pammer, David Kendler, Sebastien Rizzo, Ivo Valter, Rachel B Wagman, Xiang Yin, Susan V Yue, Georges Boivin

**Affiliations:** 1 Department of Pathology and Cell Biology, Columbia University, New York, New York; 2 Helen Hayes Hospital, West Haverstraw, New York; 3 Division of Rheumatology, Faculty of Medicine, Laval University and CHU de Quebec Research Centre, Quebec City, Quebec, Canada; 4 Department for Endocrinology and Diabetes, Medical University Graz, Graz, Austria; 5 Department of Medicine (Endocrinology), University of British Columbia, Vancouver, British Columbia, Canada; 6 Bone and Chronic Diseases, INSERM, UMR 1033, Univ Lyon, Université Claude Bernard Lyon 1, Lyon, France; 7 Center for Clinical and Basic Research, Tallinn, Estonia; 8 Clinical Development, Amgen Inc., Thousand Oaks, California

## Abstract

**Context:**

Denosumab is a potent antiresorptive agent that reduces fractures in postmenopausal women with osteoporosis.

**Objective:**

Determine effects of up to 10 years of denosumab on bone histology, remodeling, and matrix mineralization characteristics.

**Design and Setting:**

International, multicenter, randomized, double-blind trial [Fracture Reduction Evaluation of Denosumab in Osteoporosis Every 6 Months (FREEDOM)] with a long-term open-label extension.

**Patients:**

Postmenopausal women with osteoporosis (92 women in FREEDOM, 46 in extension) who provided iliac bone biopsies, including 11 who provided biopsies at multiple time points.

**Interventions:**

FREEDOM subjects were randomized 1:1 to subcutaneous denosumab 60 mg or placebo every 6 months for 3 years. Long-term extension subjects continued receiving denosumab, open-label, for 7 additional years.

**Outcomes:**

Bone histology, histomorphometry, matrix mineralization.

**Results:**

Ten-year denosumab biopsies showed normal histology. Bone histomorphometry indicated normal bone structure and reduced bone remodeling after 10 years of denosumab, similar to levels after 2 and/or 3 and 5 years of denosumab. The degree of mineralization of bone was increased and mineralization heterogeneity was reduced in the denosumab years 2/3 group vs placebo. Changes in these mineralization variables progressed from years 2/3 to year 5 of denosumab, but not thereafter.

**Conclusions:**

Denosumab for 2/3, 5, and 10 years was associated with normal histology, low bone remodeling rate, increased matrix mineralization, and lower mineralization heterogeneity compared with placebo. These variables were unchanged from year 5 to year 10. These data, in combination with the maintenance of low fracture rates for up to 10 years as previously reported with denosumab therapy, suggest that strong, prolonged remodeling inhibition does not impair bone strength.

## Teaser

After up to 10 years of denosumab therapy, iliac bone biopsies from postmenopausal women showed normal histology, reduced bone remodeling, and increased matrix mineralization that plateaued by year 5.

Denosumab is a potent antiresorptive agent that inhibits RANKL, an essential cytokine for osteoclast formation, activity, and survival (1). The pivotal 3-year placebo-controlled Fracture Reduction Evaluation of Denosumab in Osteoporosis Every 6 Months (FREEDOM) fracture trial in postmenopausal women with osteoporosis showed that denosumab increased bone mineral density (BMD), decreased biochemical markers of bone remodeling, and reduced the risk of new vertebral, nonvertebral, and hip fractures (2). Bone histomorphometry data from iliac crest biopsies collected at years 2 and 3 of FREEDOM indicated marked reductions in bone remodeling parameters with denosumab, with a majority of biopsies exhibiting no fluorochrome labeling in cancellous bone (3). These findings are consistent with denosumab’s mechanism of action; rapid and strong osteoclast inhibition leads to reduced remodeling activation, and most remodeling sites that were active when denosumab therapy was initiated would refill with mineralized matrix before the scheduled administration of fluorochrome labeling agents that are used to identify sites of active bone formation (4).

The open-label FREEDOM Extension study showed that years 5 to 10 of denosumab therapy were associated with further increases in BMD at the lumbar spine, total hip, and femoral neck; persistently low levels of biochemical and histomorphometric bone remodeling variables; and continued low rates of vertebral, hip, and nonvertebral fractures (5–9). This association between very low bone remodeling rates and reduced fracture risk with denosumab is consistent with nonclinical bone quality studies showing that denosumab increased vertebral, hip, and long bone strength, with the greatest bone strength consistently associated with the lowest levels of bone remodeling and fluorochrome labeling (4, 10).

These results notwithstanding, concerns have been raised that long-term administration of potent antiresorptive agents, including denosumab, may lead to “oversuppression” of bone remodeling that results in impaired bone matrix quality and strength (11–13). One hypothetical mechanism by which oversuppression could impair bone biomechanical properties is through microdamage accumulation (14), although experimental evidence suggests that this mechanism is not an apparent concern with denosumab. OPG-Fc, a RANKL inhibitor with a similar mechanism of action as denosumab (15), significantly reduced microdamage levels in normal and fatigue-damaged bone (16). Although greater microdamage levels are sometimes associated with lower bone toughness (17), long-term denosumab administration did not reduce toughness in nonhuman primates, despite near-total inhibition of bone remodeling (10). Another proposed mechanism by which oversuppression could potentially impair bone biomechanics is by altering matrix mineralization (14). Antiresorptive agents, including bisphosphonates (BPs) and RANKL inhibitors (18–22), increase the degree of mineralization of bone (DMB) by affording bone remodeling units more time to mineralize (18). This effect of antiresorptives also typically leads to reductions in the heterogeneity of mineralization within bone, because more bone regions are able to achieve a higher degree of mineralization (19–21, 23). The increases in DMB that result from antiresorptive therapy contribute to gains in BMD and may also contribute to reductions in fracture risk by increasing bone matrix strength and stiffness (18, 19, 24–26). However, it has also been postulated that excessive increases in bone matrix mineralization (sometimes referred to as “hypermineralization”) and/or excessive reductions in mineralization heterogeneity could lead to bone brittleness and skeletal fragility (27–29). There is no operational definition of hypermineralization or insufficient heterogeneity, and there is little experimental evidence that antiresorptive agents can alter mineralization characteristics to the point of increasing skeletal fragility. Substantial to near-total inhibition of bone remodeling in animals treated with denosumab or other RANKL inhibitors was accompanied by increases in matrix mineralization and/or reductions in mineralization heterogeneity; these changes were associated with improvements in bone structural strength, without any impairments in bone material properties (21–23). However, several evidence gaps remain in this area of research, including a paucity of data on the long-term effects of denosumab or other antiresorptive agents on matrix mineralization variables, and minimal data on associations between treatment-related changes in matrix mineralization and long-term fracture outcomes.

Long- and short-term data on the effects of denosumab on matrix mineralization may be of interest because of denosumab’s rapid and strong antiresorptive effects throughout the skeleton, including cancellous and cortical compartments (3, 4). The FREEDOM trial and its extension provided a unique opportunity to assess (1) the degree to which human bone matrix can increase its mineralization and decrease its mineralization heterogeneity as a result of denosumab treatment and (2) the potential implications of such changes on fracture rates and skeletal adverse events over an extended period of uninterrupted denosumab treatment. The current analyses from FREEDOM and its extension include bone histology, dynamic and static bone histomorphometry, and bone matrix mineralization characteristics with up to 10 years of denosumab therapy. These results are interpreted and discussed in the context of recently published data on fracture rates and other bone safety parameters in the FREEDOM extension study with up to 10 years of denosumab treatment (9).

## Subjects and Methods

### Study subjects

Subjects included in this study were enrolled in the FREEDOM trial and its extension, details of which have been previously described (2, 6). Briefly, FREEDOM enrolled 7808 women aged 60 to 91 years with median BMD T scores of –2.9 at the lumbar spine and –1.9 at the total hip. All women who completed FREEDOM and did not miss more than one dose of study drug were eligible to enter the extension. Subjects were eligible to enroll in the bone biopsy substudy if they were enrolled at a clinical trial center that was participating in the bone biopsy substudy and had no sensitivity to tetracycline or its derivatives.

### Study design

FREEDOM was a 3-year international, randomized, double-blind, placebo-controlled trial in postmenopausal women with osteoporosis (Supplemental Fig. 1) (2). Subjects received subcutaneous denosumab 60 mg or placebo every 6 months for 3 years, along with daily calcium (≥1000 mg) and vitamin D (≥400 IU) supplementation. All subjects enrolled in the FREEDOM extension were to receive open-label denosumab 60 mg every 6 months for 7 additional years. The current extension data are limited to subjects who received denosumab during FREEDOM (referred to as the long-term group), and do not include subjects who received placebo during FREEDOM (the cross-over group). Subsets of subjects underwent transiliac bone biopsies at year 2 or 3 in FREEDOM (encompassing 2 or 3 years of placebo or denosumab) and/or at year 2 or year 7 of the extension (encompassing 5 or 10 years of denosumab for subjects in the long-term group). Eleven subjects in the long-term denosumab group provided biopsies at more than one time point: one subject for denosumab year 2 and year 3; three subjects for denosumab year 3 and year 5; two subjects for denosumab year 2, year 3, and year 5; one subject for denosumab year 3, year 5, and year 10; one subject for denosumab year 3 and year 10; and three subjects for denosumab year 5 and year 10. Five subjects in the placebo group provided biopsies at year 2 and year 3. Only the year 3 data were used for subjects who had both year 2 and year 3 biopsy samples. These sequential biopsies, and a few biopsies that were not evaluable for all end points, explain occasional differences among numbers of subjects, samples, and observations.

### Bone biopsy procedures

The methodology for bone biopsy procedure and analyses was similar to previously described approaches (3). Briefly, bone biopsies were obtained from the iliac crest near the end of the 6-monthly dosing interval, within 56 days of the year 2 and/or year 3 visit (for subjects in FREEDOM) or year 5 and/or year 10 visits (year 2 and year 7 of the extension, for the long-term group). A standard double-labeling procedure was used to identify sites undergoing primary mineralization of newly formed bone, as previously described (3). Briefly, tetracycline was administered on 3 successive days, followed 14 days later by the administration of demeclocycline on three successive days, followed by biopsy 5 to 14 days after the last demeclocycline dose. Urine samples for tetracycline measurements were collected within 24 hours of the last dose of the first tetracycline labeling period to confirm compliance. Bone biopsies were obtained from the anterior iliac crest using a Bordier/Meunier or Rochester-type trephine with internal diameter of 7 to 8 mm. Specimens were fixed and shipped in 70% ethanol and then dehydrated and embedded undecalcified in glycol methylmethacrylate (GMMA).

### Bone histology and histomorphometry analyses

GMMA-embedded biopsy samples were sectioned at a 5-μm thickness at a central facility (Mayo Clinic, Rochester, MN) and mounted unstained for analyses of tetracycline labels under fluorescent microscopy. If no labels were present, a label search was performed at 5-μm intervals, as previously described (3). If fluorescent labels were present, adjacent sections were stained with toluidine blue or hematoxylin and eosin for qualitative analysis by a hematopathologist, and with Goldner trichrome stain for histomorphometric analyses of static parameters. Osteomeasure was used for histomorphometric analyses, using American Society for Bone and Mineral Research nomenclature (30). Where single fluorochrome labels were identified without evident double labels, a mineral apposition rate of 0.3 μm was imputed, per American Society for Bone and Mineral Research guidelines (30).

### Bone matrix mineralization analyses

GMMA-embedded bone biopsy samples were cross-sectioned at 150-μm thickness and thinned to 100 ± 1 μm thickness by manual grinding between a frosted glass plate and a frosted glass slide using silicon carbide powder (Escil, Chassieu, France). Sections were then polished using a 1 μm alumina suspension (Escil) and cleaned with an ultrasonic device (Elma, Singen, Germany). Section thickness was measured with a precision micrometric thickness comparator (precision of 1 μm; Compac, Geneva, Switzerland). Bone sections were analyzed for matrix mineralization by digitized quantitative microradiography in a blinded manner, using code from a MATLAB program, as previously described (31). With this method, quantitative X-ray absorption by bone tissue is reflected in grayscale values that are converted at the pixel level into a DMB, expressed in grams of mineral per cm^3^ of bone tissue. DMB reflects the density of hydroxyapatite, the mineral component of bone matrix. This conversion was based on a calibration curve generated from an aluminum reference system with a known absorption coefficient. Regions with lower matrix mineralization have a darker grayscale appearance compared with clearer, more highly mineralized regions. Five regions of bone were analyzed: the cancellous region, the total cortical region (endocortical plus periosteal combined), the endocortical subregion, the periosteal subregion, and the total bone region (cancellous plus cortical). In most cases, both cortices were analyzed, and results for each cortex were averaged. DMB was measured for each bone region and subregion, as was the heterogeneity index (HI), which reflects the width of the DMB distribution curve at one-half of its maximum height (25, 31). Limited data on total bone DMB and HI for the denosumab years 2/3, 5, and 10 groups were reported previously (9). Iliac crest bone biopsies were also obtained from 42 nonosteoporotic untreated premenopausal women (mean age, 33.4 years; SD, 4.8 years; range, 25 to 41 years) from a previously described cohort (32, 33). These patients served as a premenopausal reference range for matrix mineralization analyses of the cancellous, cortical, and total bone compartments; the endocortical and periosteal subregions of this reference group were not analyzed.

### Statistical analyses

For subjects who had both year 2 and year 3 biopsy samples in FREEDOM, only the year 3 samples were included in the calculation of group statistics. Comparisons for histology and histomorphometric data between FREEDOM placebo and FREEDOM denosumab data at years 2 and 3 combined have been published (3). For the histomorphometric parameters and bone matrix mineralization variables (DMB and HI), pairwise comparisons were performed among the four treatment groups (placebo years 2/3, denosumab years 2/3, denosumab year 5, and denosumab year 10). For DMB and HI, each of the four treatment groups was also compared with a premenopausal reference group. Two-sided Wilcoxon rank sum test was used for all comparisons between two groups without multiplicity adjustment.

## Results

There were 21 and 22 biopsies available for histomorphometry and qualitative histology (respectively) for subjects from the long-term arm of FREEDOM extension with 10 years of denosumab exposure. Bone biopsy samples from 72 women in FREEDOM (30 placebo and 42 denosumab subjects at year 2 or 3), and 28 and 21 women in the extension who had received denosumab for a total of 5 and/or 10 years, respectively, were evaluated for matrix mineralization; four of these subjects provided more than one biopsy, and one biopsy was not evaluable for mineralization analyses. Baseline characteristics were similar for women in FREEDOM, the histology and histomorphometry substudy, and the bone matrix mineralization substudy (Supplemental Table 1).

**Figure 1.**
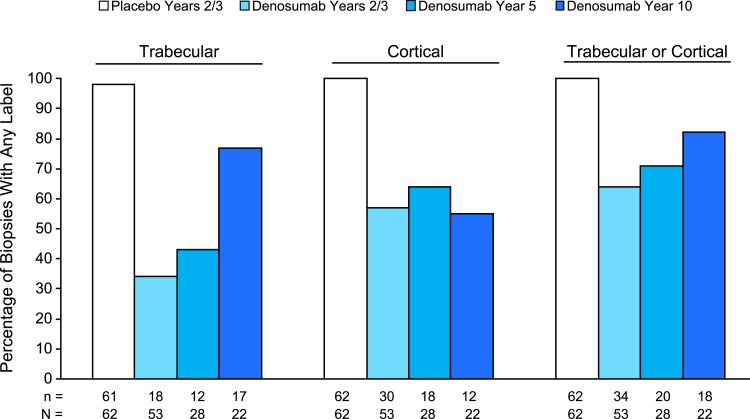
Percentage of bone biopsies with any fluorochrome on trabecular, cortical, and trabecular or cortical bone. n, number of biopsies with any label; N, number of evaluable biopsies.

Histology data for the FREEDOM biopsies (placebo and denosumab years 2/3) and the denosumab year 5 biopsies showed no adverse histopathological findings (Table 1), as previously described (3, 5). Similarly, all 22 of the denosumab year 10 biopsies evaluated for histology showed normally mineralized lamellar bone, with no evidence of pathological findings including osteomalacia, woven bone, or marrow fibrosis (Table 1). The percentage of samples with any fluorochrome labeling of trabecular bone was observed to increase over time in the denosumab samples, from 34% at years 2/3 to 43% at year 5 to 77% at year 10 (Fig. 1). Cortical labeling was evident in most denosumab samples at all three time points, with no meaningful changes over time. Double fluorochrome labeling of trabecular or cortical bone was found in 7 (32%) denosumab year 10 samples (Supplemental Table 2). Additional details on fluorochrome labeling status are provided in Supplemental Table 2.

**Table 1.** Bone Histology and Histopathology

	FREEDOM		Extension	
	Placebo Year 2 and/or 3	Denosumab Year 2 and/or 3	Denosumab Year 5	Denosumab Year 10
	N = 45*^a^*	N = 47*^a^*	N = 28*^b^*	N = 22*^b^*
Evaluable biopsies*^c^*	62	53	28	22
Normal lamellar bone, n (%)	62 (100)	53 (100)	28 (100)	22 (100)
Normal mineralization, n (%)	62 (100)	53 (100)	28 (100)	22 (100)
Present osteoid, n (%)	62 (100)	48 (91)	23 (82)	18 (82)
No visible osteoid, n (%)	0 (0)	5 (9.4)	5 (17.9)	4 (18.2)
Osteomalacia, n	0	0	0	0
Marrow fibrosis, n	0	0	0	0
Woven bone, n	0	0	0	0

^a^ Number of subjects who enrolled in the FREEDOM bone biopsy substudy, received ≥1 dose of investigational product during FREEDOM, and had an evaluable biopsy at year 2 or year 3.

^b^ Number of subjects who enrolled in the extension bone biopsy substudy, received ≥1 dose of investigational product during the extension, and had an evaluable biopsy at the time point(s) of interest.

^c^ Number of evaluable biopsies, which serves as the denominator for percentage values in parentheses; some subjects had ≥1 evaluable biopsy during the FREEDOM trial.

**Figure 2.**
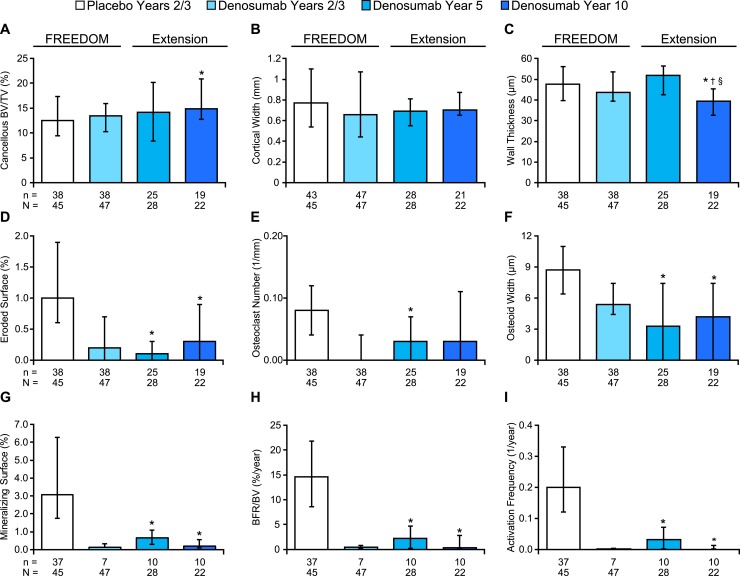
Bone histomorphometry results for iliac crest bone biopsies from FREEDOM and the extension. All parameters except cortical width were obtained from cancellous bone. Data represent median and interquartile range, n = number of subjects with observed data; N = number of randomized subjects who enrolled in the bone biopsy substudy who received at least 1 dose of investigational product during FREEDOM (for the FREEDOM groups) and during extension (for the extension groups), and had at least one evaluable biopsy. *P < 0.05 vs placebo years 2/3; ^†^*P* < 0.05 vs denosumab year 2/3; ^§^*P* < 0.05 vs denosumab year 5, by two-sided Wilcoxon rank-sum test. BFR, bone formation rate; TV, tissue volume.

Histomorphometric analyses for FREEDOM and year 2 of the extension (5 years of denosumab) were presented previously (3, 5) and are included in Fig. 2 and Supplemental Table 3 for context. Year 10 histomorphometric data showed that the antiresorptive effect of denosumab was maintained over time, with no significant differences in cancellous eroded surface or osteoclast number compared with values for denosumab subjects at years 2/3 or year 5 (Fig. 2). Cancellous bone volume per tissue volume (BV/TV) was higher and eroded surface and osteoid width were lower in the year 10 samples compared with the years 2/3 placebo samples, with no significant differences for these variables between denosumab years 2/3, year 5, or year 10 (Fig. 2). Cancellous wall thickness was significantly lower in the denosumab year 10 group compared with the other three groups (Fig. 2). Cortical width remained similar in the denosumab samples over time, as did trabecular thickness (Fig. 2; Supplemental Table 3). Trabecular number was higher and trabecular separation was lower in the denosumab year 10 samples compared with placebo, denosumab years 2/3, and denosumab year 5 (Supplemental Table 3). The dynamic parameters mineralizing surface, mineral apposition rate, bone formation rate per bone volume, and activation frequency (9) were significantly lower in the year 10 denosumab group compared with placebo, but similar to values from the denosumab years 2/3 and year 5 groups (Fig. 2; Supplemental Table 3). Osteoid surface was lower in the year 5 and year 10 denosumab samples compared with the years 2/3 denosumab samples, whereas the percentages of denosumab biopsies that showed any osteoid remained similar over time (range, 82% to 91%) (Supplemental Table 4). Several samples that lacked visible osteoid did display tetracycline labels, indicating that the absence of visible osteoid does not necessarily mean lack of bone formation (Supplemental Table 4).

**Figure 3.**
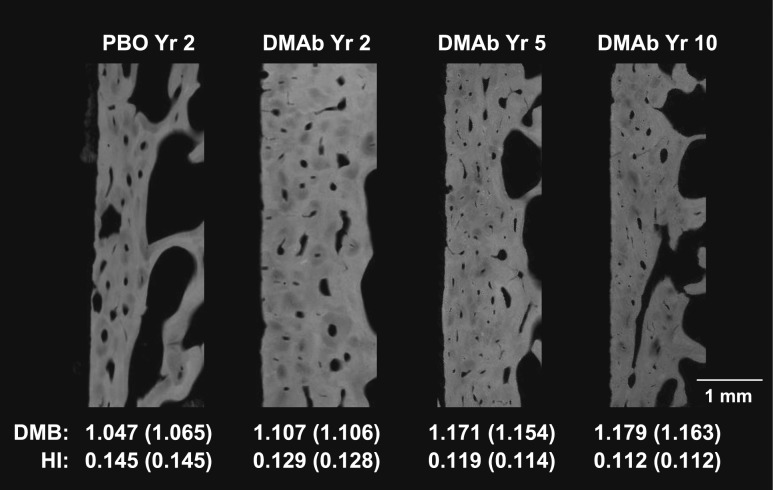
Digitized microradiographs of single cortices of iliac crest bone biopsies representative of placebo (PBO) year 2 group and denosumab (DMAb) years 2, 5, and 10. Samples were selected based on cortical DMB and HI values (bottom) similar to their respective group’s median values (in parentheses). For the year 2 PBO and DMAb samples, the group median values (in parentheses) represent values for years 2 and 3 combined.

**Figure 4.**
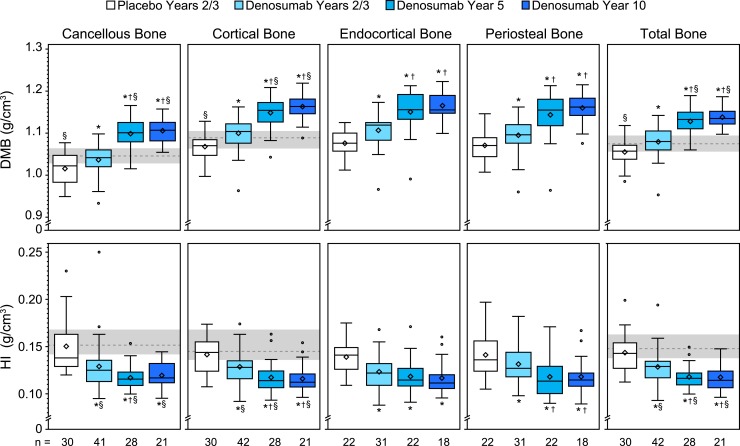
DMB and HI for iliac crest bone biopsies. Note that the *y*-axis scales are truncated. For the box-and-whisker plots, the box’s lower bound represents the first quartile (Q1), its upper bound represents the third quartile (Q3), the line is the median, the diamond is the mean, and the circles are outliers. The gray bands with dashed lines represent the interquartile range (Q1–Q3) and median value, respectively, for the premenopausal reference group (n = 42), from which endocortical and periosteal subcompartment mineralization data were not obtained. *P < 0.05 vs placebo years 2/3, ^†^*P* < 0.05 vs denosumab years 2/3, ^§^*P* < 0.05 vs premenopausal reference group, by two-sided Wilcoxon rank-sum test for between-group comparisons. n = number of subjects with observed data.

Representative images depicting the degree and heterogeneity of matrix mineralization are shown in Fig. 3, which highlights single cortices from digitized microradiographs of samples that have cortical DMB and HI values similar to their group median. The heterogeneity of mineralization is visually reflected by the proportion and distribution of darker, less-mineralized osteons relative to lighter, more-mineralized osteons and interstitial bone. Quantified HI and DMB results show that between-group differences for the total bone region were generally reflective of changes observed in the cancellous, cortical, endocortical, and periosteal subregions (Fig. 4). DMB was significantly greater in the denosumab years 2/3 vs placebo group at years 2/3 for the total bone and for each subregion. The year 5 denosumab samples showed significantly greater DMB for total bone and for each subregion compared with the years 2/3 denosumab and placebo samples. For denosumab year 5, the median DMB value for total bone was 1.132 g/cm^3^ [interquartile range (IQR), 1.110 to 1.150], which was 7.3% higher compared with the median value of the placebo group (1.055 g/cm^3^; IQR, 1.034 to 1.070). DMB for total bone in the year 10 denosumab group (1.135 g/cm^3^; IQR, 1.122 to 1.152) was similar to that of the year 5 group, and significantly higher (by 7.6%) compared with the placebo group (*P* < 0.05). Compared with the premenopausal reference group, DMB values for total, cortical, and cancellous bone were significantly lower in the placebo group and significantly higher in the denosumab year 5 and year 10 groups (Fig. 4).

HI for the total bone and for all subregions was significantly lower in the years 2/3 denosumab samples vs placebo (Fig. 4). The year 5 denosumab samples had significantly lower HI for total bone and for all subregions except endocortical compared with the years 2/3 denosumab samples. At year 5, median HI for total bone (0.116 g/cm^3^; IQR, 0.110 to 0.122) was 19.4% lower compared with years 2/3 placebo controls (0.144 g/cm^3^; IQR, 0.135 to 0.157). The group median value for HI in the year 10 denosumab group (0.114 g/cm^3^; IQR, 0.106 to 0.124) was similar to that for the denosumab year 5 group (0.116 g/cm^3^; IQR, 0.110 to 0.122), suggesting a steady state of heterogeneity was established by approximately year 5. Compared with the premenopausal reference group, HI values for total, cortical, and cancellous bone were significantly lower in the denosumab years 2/3, 5, and 10 groups (Fig. 4).

**Figure 5.**
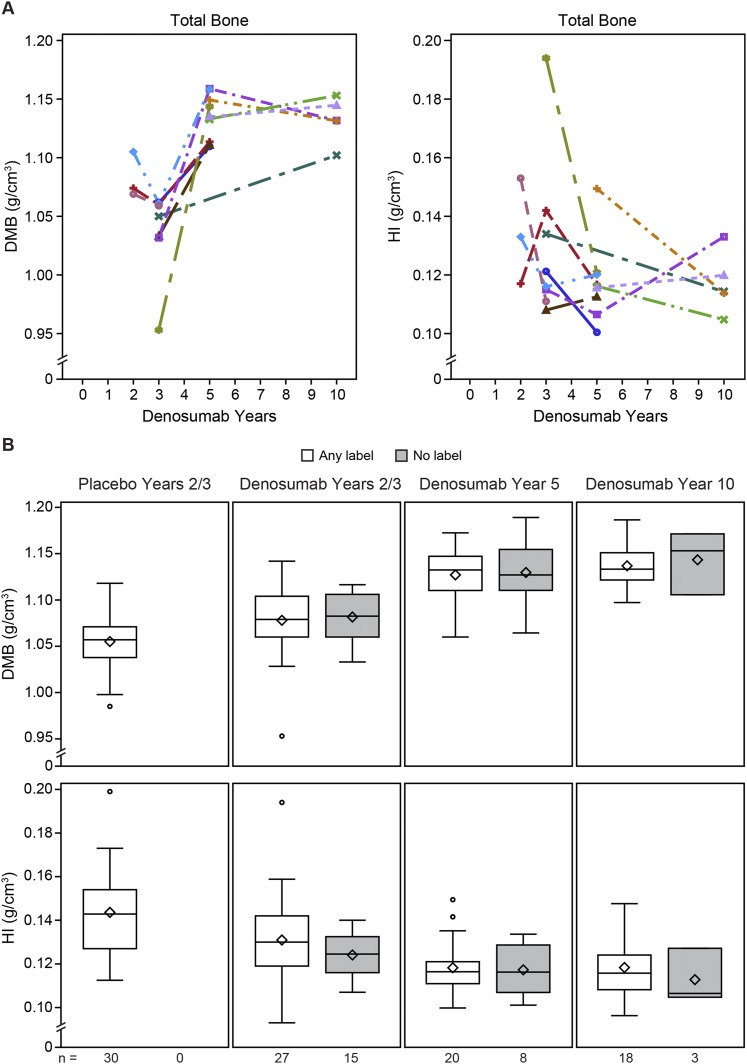
Additional DMB and HI results for denosumab-treated subjects. Note that the *x*-axis scales are truncated. (A) Results for sequential biopsies from 11 denosumab-treated subjects that provided serial bone biopsy samples. (B) DMB and HI results in total bone for subjects with and without fluorochrome labels. The box-and-whisker plots and n values are explained in the Fig 4. legend.

For the FREEDOM and extension studies, changes in DMB and HI were similar for individual subjects from whom multiple sequential biopsies were obtained vs between-group differences observed for the larger cross-sectional sample set, which mostly comprised nonsequential biopsies (Fig. 5A). DMB and HI values were similar in denosumab subjects without fluorochrome labels compared with those with labels (Fig. 5B).

## Discussion

This study showed that normal bone histology was maintained through 10 years of denosumab therapy in postmenopausal women with osteoporosis from the FREEDOM long-term extension bone biopsy substudy. These findings, including a lack of evidence for woven bone, marrow fibrosis, or impaired matrix mineralization, are similar to previous analyses conducted at year 5 of the FREEDOM long-term extension study (5). Bone histomorphometry findings for denosumab years 2/3, year 5, and year 10 are consistent with the mechanism of action of denosumab, which potently inhibits bone resorption and remodeling and increases bone mass and strength (4). The year 10 data show few differences compared with histomorphometry data for years 2/3 and year 5 of denosumab therapy (3, 5); these differences include higher trabecular number and lower trabecular spacing, although the year 10 findings were largely based on cross-sectional comparisons to earlier time points. There were only five sequential biopsies that bridged year 10 with earlier time points, which are too few to make meaningful conclusions about changes over time. The percentage of biopsies with cancellous fluorochrome labels was also observed to be higher at year 10 compared with earlier denosumab time points, but dynamic histomorphometry otherwise indicated very low levels of bone turnover at year 10, indicating that the strong antiremodeling effects of denosumab were maintained over this treatment duration. Systemic bone turnover markers also remained substantially reduced through 10 years of denosumab (9), although modest release of this inhibition tends to occur toward the end of the 6-month denosumab dosing interval (34). Recognizing that iliac crest bone biopsies represent a small, non–weight-bearing sample of the entire skeleton, these findings collectively suggest that denosumab markedly inhibits bone remodeling throughout much of the skeleton, an effect that is associated with persistently low fracture rates through at least a decade of therapy (9).

Concerns have been raised that long-term administration of potent remodeling inhibitors might have deleterious effects on bone strength, potentially manifesting as rare atypical femur fractures (AFFs) (11, 13, 28, 35). These concerns, originally based on associations observed in BP studies, may also apply to denosumab, which has even greater antiremodeling effects (36, 37). Remodeling inhibitors may influence bone strength through changes in bone matrix mineralization, although it remains unclear whether such effects are biomechanically favorable or unfavorable (13, 14, 25, 38). The current study used microradiography to assess temporal changes in bone matrix mineralization characteristics during 10 years of denosumab therapy. Microradiography-based matrix mineralization data were previously shown to correlate with tissue-level bone strength (39). The digital microradiography method used in the current study was validated and used in previous clinical studies (31, 40, 41). As expected for a potent remodeling inhibitor, denosumab increased the overall degree of mineralization (*i.e.,* DMB), and reduced the heterogeneity of mineralization (HI), compared with placebo. These DMB and HI changes were progressive with up to 5 years of denosumab therapy, with minimal, nonsignificant changes thereafter.

The FREEDOM long-term extension population may have experienced the greatest overall bone remodeling inhibition of any group of treated postmenopausal women studied to date. Denosumab does not incorporate into bone matrix (15), which eliminates the potential for direct effects on mineralization characteristics or matrix material properties that may potentially arise from skeletal uptake of BPs (42). As such, the current changes in DMB and HI may be among the largest achieved through remodeling inhibition in a clinical trial setting, presenting a powerful opportunity to address true relationships between remodeling, matrix mineralization, and fracture risk. Previous quantitative backscattered electron imaging (qBEI) data showed that the degree of cancellous bone matrix mineralization in one study of BP-treated postmenopausal women with osteoporosis exceeded levels found in a skeletally healthy reference group (20), whereas in other studies, the heterogeneity of mineralization was similar in BP-treated subjects compared with this same reference group (20, 43). In the current study, DMB and HI values in the year 5 and 10 denosumab samples were both significantly different vs a premenopausal reference group, which further suggests a sizable treatment effect on the degree and heterogeneity of mineralization. It is therefore interesting to note that the overall study population from which the current biopsy subset was drawn (2343 women in the long-term arm of the extension study, 1343 of whom completed the study) showed persistently low rates of new vertebral, nonvertebral, and hip fractures through year 10 (9), with rates of nonvertebral fractures being lower during the extension compared with the rate for denosumab subjects during FREEDOM (44). Those findings indicate that the duration and degree of DMB and HI changes achieved here do not weaken bone or increase fracture risk at the population level. One subject from the long-term arm of the extension study (*i.e.,* the group receiving denosumab since the beginning of FREEDOM) experienced an event consistent with the definition of AFF after 7 total years of denosumab (9), but this subject was not part of the biopsy substudy, and her bone matrix mineralization characteristics remain unknown. Other recent data indicated that bone matrix adjacent to AFF sites in BP-treated women had higher mineralization, as suggested by a greater mineral-to-organic-matrix ratio, compared with fracture samples from BP-treated and untreated women with typical femur fractures. However, the same samples showed that qBEI-derived variables that correspond to DMB (*i.e.,* calcium mean) and HI (*i.e.,* calcium width) were similar at AFF vs non-AFF sites from long-term BP users, and were also similar at AFF sites of BP users vs nonfractured femoral bone tissue from non-BP users (28). The current results, combined with long-term denosumab fracture data (9) and long-term denosumab bone quality data (10), suggest that the changes in matrix mineralization characteristics resulting from up to 10 years of denosumab treatment have favorable effects on bone structural strength. Similarly, the low levels or absence of fluorochrome labeling in bone biopsies do not appear to carry negative implications for bone strength in the current population and is likely a reflection of efficacy (4). Indeed, there was no difference in mineralization variables between subjects that had detectable fluorochrome labels and those that did not.

There are limited data on the effects of antiresorptives on matrix mineralization beyond 3 years, with one BP study reporting that changes in the degree and heterogeneity of mineralization did not progress from year 3 to year 5 of treatment (19), and another showing no progressive changes in mineralization characteristics between years 2/3 and year 10 of treatment (43). Evidence of progressive changes in DMB and HI between denosumab years 2/3 and year 5 may be a unique finding among antiresorptive therapies and may have clinical implications. First, these findings suggest that the total remodeling period in these denosumab subjects, including secondary mineralization, is ∼5 years. A study in alendronate-treated postmenopausal women indicated that a new steady state of higher and less heterogeneous matrix mineralization was achieved within 2 to 3 years (18). This may indicate that secondary mineralization of preexisting remodeling units reached completion within 3 years after initiating alendronate, although ongoing residual remodeling may have influenced those results. Compared with alendronate, denosumab causes more rapid and more substantial inhibition of bone remodeling; bone resorption markers were greatly reduced within 3 days of initiating denosumab therapy (34), suggesting that changes in matrix mineralization begin within the first week of treatment. Based on that early trigger, and on observed changes in DMB and HI up to year 5, the time required to refill existing remodeling spaces and complete their primary and secondary mineralization appears to be ∼5 years. It is unclear whether this 5-year duration is unique to denosumab-treated postmenopausal women or whether it might apply to other populations as well. Second, these findings imply that increased matrix mineralization may contribute to progressive BMD gains with denosumab therapy for up to ∼5 years, but perhaps not thereafter. Denosumab causes continued BMD gains between year 5 and year 10 of treatment (9), and those gains may result from mineralization-independent phenomena, perhaps including modeling-based bone formation (MBBF). MBBF persisted in femoral neck of denosumab-treated ovariectomized cynomolgus monkeys despite continued strong inhibition of resorption and remodeling, and cancellous MBBF was significantly increased in iliac crest bone biopsies from denosumab-treated postmenopausal women (45, 46). A third implication of the mineralization findings relates to overall bone strength. Matrix mineralization and its tissue-level strength are strongly related (26), and increased matrix mineralization and/or reduced mineralization heterogeneity with denosumab and other RANKL inhibitors was associated with increased bone strength in animals (21–23). Those findings, and the current data, imply that changes in matrix mineralization may lead to progressive increases in bone strength for up to ∼5 years of denosumab therapy, which would represent the minimum duration of treatment for achieving the full biomechanical benefits conferred by denosumab’s antiremodeling effect.

The reductions in HI in the 5- and 10-year samples were highly statistically significant vs placebo, and HI was also significantly lower for all denosumab treatment durations compared with a premenopausal reference range, yet the absolute reductions in HI compared with placebo seem modest in light of the degree and duration of remodeling inhibition associated with denosumab. As such, this study does not provide clear insights into the biomechanical implications of highly and homogenously mineralized matrix. It is reasonable to suspect that such a scenario, were it achievable, might represent a truly hypermineralized state characterized by inferior biomechanical properties that manifest through mechanisms previously proposed (13, 14, 28). As mentioned, biochemical markers of bone turnover tend to show some release of inhibition toward the end of the 6-monthly denosumab dosing interval, and we cannot exclude the possibility that “breakthrough” remodeling contributed to the lack of hypermineralization. However, these biopsies were collected near the end of the denosumab dosing interval, and dynamic histomorphometry nonetheless indicated very low remodeling rates in all denosumab groups, with activation frequencies at or near zero. These data suggest that the lack of hypermineralization in the year 10 biopsies is not for want of greater osteoclast inhibition, and may imply that factors besides remodeling activation could be functioning as self-regulatory mechanisms within bone that maintain key mineralization characteristics within biomechanically acceptable ranges. In support of this hypothesis, the remodeling period (47) and the rate of secondary matrix mineralization (39) both increase substantially with age, and antiresorptive therapies including denosumab can also increase the formation period (3). Each of these changes may serve to limit the potential for extreme increases in DMB and extreme decreases in HI when osteoclasts become inhibited.

This study has several limitations, including a relatively small number of bone biopsies, which limits the ability to compare the results with specific patient outcomes, including fragility fractures and rare safety events such as AFF and osteonecrosis of the jaw. There were few paired (*i.e.,* sequential) bone biopsies, although observed changes in matrix mineralization parameters for the paired biopsy subset generally align with intergroup differences observed for the entire sample set, which mostly comprised unpaired biopsies. The lack of placebo controls in FREEDOM extension limits firm conclusions regarding the effects of denosumab on histomorphometry and matrix mineralization characteristics at years 5 and 10. Finally, the microradiography method used to assess matrix mineralization provides end points that are analogous but not identical to those provided by other methods (*e.g.,* qBEI, Raman spectroscopy), making it difficult to directly compare the current results against matrix mineralization data from some previous clinical trials of antiresorptive agents.

In summary, iliac crest bone biopsies obtained from a subset of postmenopausal women from the long-term arm of FREEDOM extension study showed (1) maintenance of normal bone histology, (2) maintenance or improvements in bone microstructural parameters, (3) a persistently low state of bone resorption and remodeling, and (4) changes in bone matrix mineralization characteristics that are consistent with denosumab’s mechanism of action as a potent remodeling inhibitor. The degree and heterogeneity of bone matrix mineralization changed with up to 5 years of denosumab and remained similar between the year 5 and year 10 denosumab biopsies. These findings, when viewed in the light of 10-year data on fracture rates and bone safety assessments from the FREEDOM extension (9), indicate that denosumab maintains a favorable efficacy and bone safety profile for 10 years of uninterrupted therapy.

## Supplementary Material

Supplemental Datas

Supplemental Figure 1

## Abbreviations:

AFF: atypical femur fracture
BMD: bone mineral density
BP: bisphosphonate
DMB: degree of mineralization of bone
FREEDOM: Fracture Reduction Evaluation of Denosumab in Osteoporosis Every 6 Months
GMMA: glycol methylmethacrylate
HI: heterogeneity index
IQR: interquartile range
MBBF: modeling-based bone formation
qBEI: quantitative backscattered electron imaging
