# Supplementary material for: Effects of Long-Term Denosumab on Bone Histomorphometry and Mineralization in Women With Postmenopausal Osteoporosis
Source: J Clin Endocrinol Metab. 2018 Apr 16;103(7):2498–509. doi: 10.1210/jc.2017-02669 (PMC6037073; doi:10.1210/jc.2017-02669)

Supplemental Table S1. Subject baseline characteristics for histology, histomorphometry, and matrix mineralization analyses upon enrollment in FREEDOM.

	FREEDOM		Histology and Histomorphometry Analysis				Bone Matrix Mineralization Analysis			
			FREEDOM		Extension		FREEDOM		Extension	
	Pbo<br>N = 3906	DMAb<br>N = 3902	Pbo<br>Year 2/3<br>n = 45	DMAb<br>Year 2/3<br>n = 47	DMAb<br>Year 5<br>n = 28	DMAb<br>Year 10<br>n = 22	Pbo<br>Year 2/3<br>n = 30	DMAb<br>Year 2/3<br>n = 42	DMAb<br>Year 5<br>n = 28	DMAb<br>Year 10<br>n = 21
Age (years)	72.3 (5.2)	72.3 (5.2)	70.0 (6.3)	72.0 (5.2)	72.5 (3.9)	68.8 (6.2)	70.2 (6.1)	72.0 (5.3)	72.5 (3.9)	69.0 (6.3)
Years since menopause	24.2 (7.5)	24.2 (7.4)	23.0 (8.1)	24.9 (7.3)	23.1 (5.3)	18.2 (8.4)	22.5 (8.3)	25.5 (7.3)	23.1 (5.3)	18.7 (8.3)
BMD T-score										
Lumbar spine	–2.8 (0.7)	–2.8 (0.7)	–2.9 (0.5)	–2.9 (0.5)	–2.9 (0.8)	–2.9 (1.2)	–2.9 (0.5)	–2.9 (0.6)	–2.9 (0.8)	–2.8 (1.3)
Total hip	–1.9 (0.8)	–1.9 (0.8)	–1.8 (0.8)	–2.0 (0.8)	–2.0 (0.8)	–1.6 (1.0)	–1.9 (0.6)	–2.0 (0.8)	–2.0 (0.8)	–1.6 (1.0)
Femoral neck	–2.2 (0.7)	–2.1 (0.7)	–2.0 (0.7)	–2.3 (0.7)	–2.2 (0.7)	–1.9 (0.6)	-2.1 (0.6)	-2.3 (0.8)	-2.2 (0.7)	-1.9 (0.7)
Serum CTX (ng/mL), median (Q1, Q3)	0.54<br>(0.38, 0.72)	0.54<br>(0.38, 0.71)	0.50<br>(0.32, 0.63)	0.47<br>(0.38, 0.63)	0.46<br>(0.37, 0.66)	0.69<br>(0.54, 0.85)	0.50<br>(0.34, 0.61)	0.51<br>(0.38, 0.63)	0.46<br>(0.37, 0.66)	0.72<br>(0.56, 0.85)
Serum P1NP (µg/mL), median (Q1, Q3)	54.16<br>(41.88, 69.34)	54.44<br>(41.44, 69.41)	50.27<br>(37.30, 60.96)	51.08<br>(40.79, 63.18)	54.28<br>(41.04, 68.59)	63.17<br>(54.28, 89.99)	51.34<br>(39.77, 60.68)	51.10<br>(40.79, 63.18)	54.04<br>(41.04, 68.59)	70.10<br>(55.39, 90.95)

Data are shown as mean (SD) unless noted otherwise. N = Number of subjects randomized at FREEDOM; n = Number of subjects who received ≥ 1 dose of investigational product and had an evaluable biopsy at the timepoint of interest.
DMAb, denosumab; Pbo, placebo; Q1, first quartile; Q3, third quartile.

Supplemental Table S2. Additional labeling status data

	Trabecular		Cortical		Trabecular or Cortical	
FREEDOM Years 2/3	Placebo	Denosumab	Placebo	Denosumab	Placebo	Denosumab
Evaluable biopsies, n	62	53	62	53	62	53
Any label	61 (98)	18 (34)	62 (100)	30 (57)	62 (100)	34 (64)
Double label	58 (94)	10 (19)	61 (98)	16 (30)	62 (100)	21 (40)
Single label only	3 (5)	8 (15)	1 (2)	14 (26)	0	13 (25)
No label	1 (2)	35 (66)	0	23 (43)	0	19 (36)
Extension Year 5		Long-term Denosumab		Long-term Denosumab		Long-term Denosumab
Evaluable biopsies, n		28		28		28
Any label		12 (43)		18 (64)		20 (71)
Double label		8 (29)		11 (39)		14 (50)
Single label only		4 (14)		7 (25)		6 (21)
No label		16 (57)		10 (36)		8 (29)
Extension Year 10		Long-term Denosumab		Long-term Denosumab		Long-term Denosumab
Evaluable biopsies, n		22		22		22
Any label		17 (77)		12 (55)		18 (82)
Double label		3 (14)		7 (32)		7 (32)
Single label only		14 (64)		5 (23)		11 (50)
No label		5 (23)		10 (45)		4 (18)

Data are shown as n (%) unless noted otherwise. n = Number of biopsies. Percentages are calculated based on number of evaluable biopsies; some subjects had ≥1 evaluable biopsy during FREEDOM and the Extension.

Supplemental Table S3. Additional bone histomorphometry data

	FREEDOM				Extension			
		Placebo Year 2/3 (N = 45)		Denosumab Year 2/3 N = 47		Denosumab Year 5 N = 28		Denosumab Year 10 N = 22
	n	Median (Q1, Q3)	*n*	Median (Q1, Q3)	*n*	Median (Q1, Q3)	*n*	Median (Q1, Q3)
Mineral apposition rate (μm/d)	37	0.750 (0.660, 0.830)	7	0.300* (0.300, 0.500)	10	0.405 (0.300, 1.050)	10	0.300* (0.300, 0.300)
Bone formation period (days)	37	113.7 (65.7, 178.2)	7	459.2* (196.5, 1581.4)	10	125.2 (40.2, 328.3)	10	16.0 (0.0, 194.0)
Trabecular thickness (μm)	38	145.790 (118.750, 165.830)	38	138.305 (121.370, 160.090)	25	148.270 (119.730, 185.790)	19	134.990 (107.350, 148.380)
Trabecular separation (μm)	38	1010.340 (749.280, 1291.160)	38	895.850 (737.620, 1033.130)	25	999.500 (718.090, 1356.700)	19	695.070*†§ (560.620, 836.550)
Trabecular number (1/mm)	38	0.875 (0.700, 1.080)	38	0.955 (0.850, 1.170)	25	0.890 (0.690, 1.060)	19	1.220*†§ (1.030, 1.410)

N = Number of subjects enrolled in bone biopsy substudy, received ≥1 dose of investigational product, and had an evaluable biopsy (excluding biopsies only evaluable for cortical width); n = Number of subjects with observed data.
Q1, first quartile; Q3, third quartile.
**P*<0.05 vs placebo years 2/3.
†*P*<0.05 vs denosumab years 2/3.
§*P*<0.05 vs denosumab year 5.

Supplemental Table S4. Osteoid surface and osteoid status

	FREEDOM		Extension	
	Placebo Year 2/3 N = 45	Denosumab Year 2/3 N = 47	Denosumab Year 5 N = 28	Denosumab Year 10 N = 22
Osteoid surface (%), median (Q1, Q3)	6.810 (3.610, 10.100)	0.385 (0.160, 1.220)*	0.140 (0.000, 0.820)†	0.140 (0.000, 0.210)†
Evaluable biopsies[a]	62	53	28	22
Present osteoid, n (%)	62 (100)	48 (91)	23 (82)	18 (82)
No visible osteoid, n	0	5	5	4
Any fluorochrome label, n				
Double label	–	1	0	1
Single label	–	2	2	2
No fluorochrome label	–	2	3	1

N = Number of subjects enrolled in bone biopsy substudy, received ≥1 dose of investigational product, and had an evaluable biopsy; n = Number of biopsies; osteoid percentages are calculated based on number evaluable biopsies.

Q1, first quartile; Q3, third quartile.

[a]Number of evaluable biopsies; some subjects had more than 1 evaluable biopsy during the FREEDOM trial.

*P<0.05 vs placebo years 2/3.

†P<0.05 vs denosumab years 2/3.

Supplemental Figure S1: Study design for the FREEDOM trial and its long-term extension. N = Number of randomized subjects who enrolled in the respective bone biopsy substudy, received at least 1 dose of investigational product during FREEDOM (for the FREEDOM groups) and during Extension (for the Extension groups), and had at least one evaluable biopsy. Circles represent bone biopsy time points.

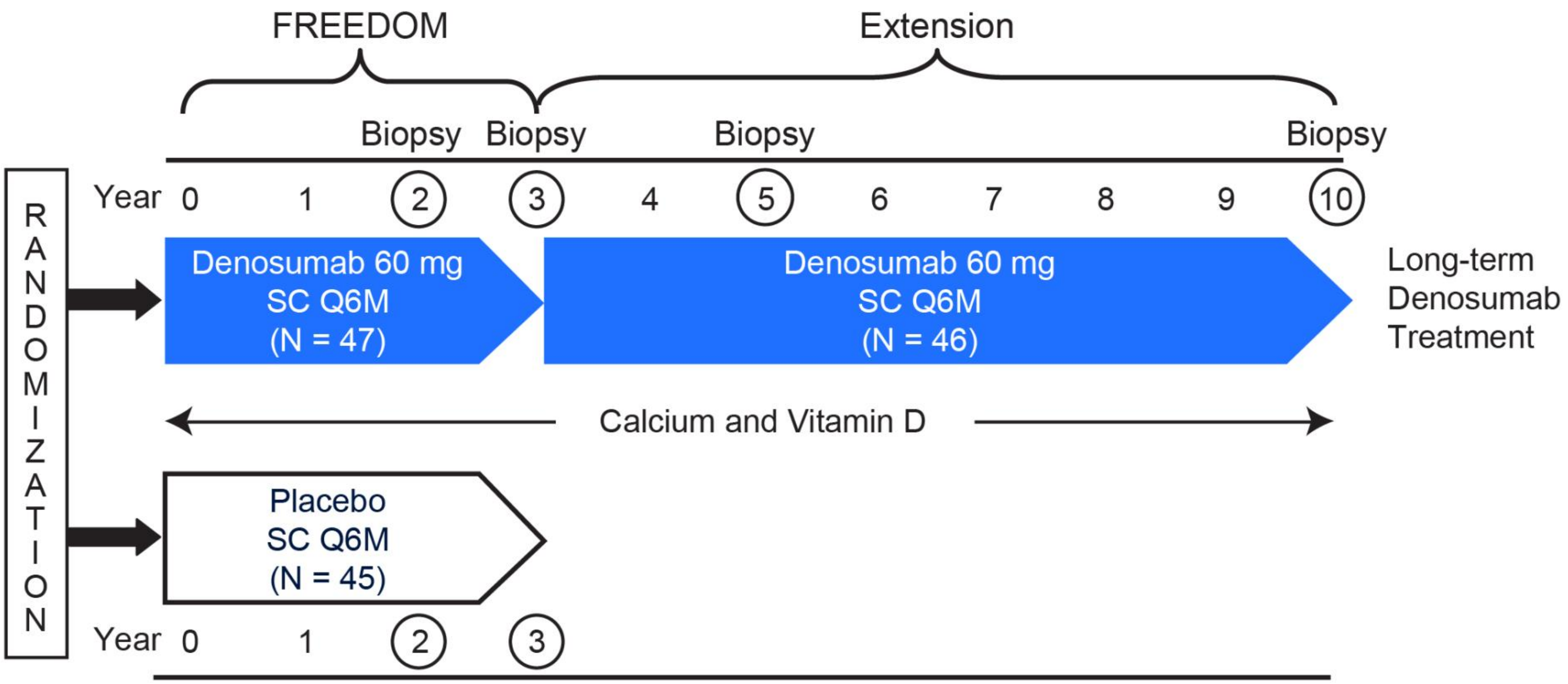

Supplement: Supplemental Datas [file jc.2017-02669.sd1.pdf]
