# Supplementary figures and images for: Effects of Long-Term Denosumab on Bone Histomorphometry and Mineralization in Women With Postmenopausal Osteoporosis
Source: J Clin Endocrinol Metab. 2018 Apr 16;103(7):2498–509. doi: 10.1210/jc.2017-02669 (PMC6037073; doi:10.1210/jc.2017-02669)

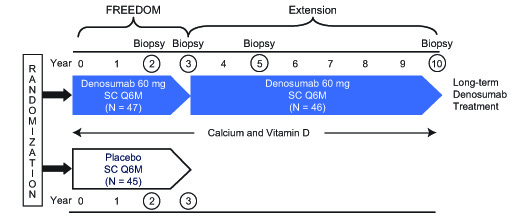

Supplement: Supplemental Figure 1 [file jc.2017-02669.sf1.jpeg]
